# Editorial: Management of Diabetes and its Complications: A Focus on Endothelial Dysfunction

**DOI:** 10.3389/fendo.2022.857983

**Published:** 2022-03-18

**Authors:** Shanhu Qiu, Jianhua Ma, Tongzhi Wu

**Affiliations:** ^1^ Department of General Practice, Zhongda Hospital; Institute of Diabetes, School of Medicine, Southeast University, Nanjing, China; ^2^ Research and Education Centre of General Practice, Zhongda Hospital, Southeast University, Nanjing, China; ^3^ Department of Endocrinology, Shenzhen People’s Hospital, The Second Clinical Medical College of Jinan University, Shenzhen, China; ^4^ Department of Endocrinology, Nanjing First Hospital, Nanjing Medical University, Nanjing, China; ^5^ Adelaide Medical School and Centre of Research Excellence (CRE) in Translating Nutritional Science to Good Health, The University of Adelaide, Adelaide, SA, Australia

**Keywords:** diabetes, endothelial function, neuropathy, retinopathy, albuminuria

Diabetes has become a demonstrably public health challenge in the world (1–3), affecting approximately 537 million adults (10.5% of the global adult population) in 2021, based on the 10th edition of the International Diabetes Federation Diabetes Atlas (2). Despite reported declines in the rates of diabetes-related macrovascular complications [e.g., myocardial infarction and ischemic stroke (4)] and microvascular complications (e.g., lower-extremity amputation and end-stage renal disease) over the past decades (5), these complications continue to contribute to the substantial demands on health services and healthcare costs. Understanding the pathogenesis of diabetes and its complications is therefore of major clinical importance to the development of targeted interventions to prevent or delay their development and/or progression and reduce the associated health and economic burden.

Endothelial dysfunction, which is characterized as an impaired or diminished ability of the endothelium to maintain vascular homeostasis (e.g., vasodilation and vascular permeability), is recognized to be an important determinant that underpins the development of diabetes and diabetes-related macrovascular and microvascular complications (6, 7). In this Research Topic of “*Management of Diabetes and its Complications: A Focus on Endothelial Dysfunction*”, we aimed to gather the latest knowledge from basic research to clinical studies relating to the pathogenesis or etiology of diabetes and its complications, with an emphasis on endothelial dysfunction, in order to shed light on potential strategies for improved management of diabetes and its complications.

Diabetic cardiomyopathy is associated with increased risk of heart failure and occurs more frequently among patients with microvascular complications than those without (8). Recent evidence suggests that injuries on cardiac microvascular endothelial cells (CMECs) might contribute to the development of diabetic cardiomyopathy (9). However, it remains unknown as to how diabetes affects CMECs at the level of cellular metabolism. The study by Zhang et al. provided some insights into this issue, showing that diabetes was associated with decreased glycolytic reserve, increased maximal respiration, and down-regulated expression of several key genes involved in the regulation of β-oxidation, tricarboxylic acid cycle, and electron transport chain, in CMECs, which were isolated from the hearts of male C57BL/6 and diabetic (db/db) mice. Moreover, diabetes may reduce the proliferation but promote the apoptosis of CMECs.

Diabetic small-fibre neuropathy is characterized by damages on small-diameter (type C and Aδ) nerve fibres and plays an essential role in the pathogenesis of foot ulceration and autonomic neuropathy (10). As an attempt to enrich the understanding of the pathophysiology of this disease, Ando et al. showed in their case-control study that corneal confocal microscopy parameters, which are considered surrogate markers of small-fibre nerve damage, were positively associated with reactive hyperaemia peripheral arterial tonometry, a measure used to assess endothelial dysfunction, in patients with type 2 diabetes. However, the conclusion that endothelial dysfunction might contribute to the development of diabetic small fibre neuropathy requires further validation in longitudinal or interventional studies.

Diabetic retinopathy is a major cause of blindness in the working-age population. Endothelial dysfunction, in particular retinal endothelial dysfunction, is recognized to be central to the development and progression of diabetic retinopathy (11). In the review paper, Gui et al. summarized current evidence on the contribution of different factors, including advanced glycosylation end products and receptors, pro-inflammatory cytokines and chemokines, and growth factors, to retinal endothelial dysfunction, and discussed their associations with the progression of diabetic retinopathy. However, only one half of the patients with advanced diabetic retinopathy (e.g., diabetic macular edema) responded fully to anti-VEGF treatment, which targets at endothelial dysfunction (12). This observation suggests that endothelial regulators other than VEGF may be involved or that pathogenic factors beyond endothelial dysfunction may also underline diabetic retinopathy.

In addition to articles focusing on diabetes-related complications and endothelial dysfunction, we had 2 additional articles in this topical collection that concerned the pathogenesis and the prevention of diabetes. The review article by Zhou et al. summarized available literature on the protective effects of Vitamin A and its derivatives on the pancreas, which covered a broad spectrum of actions on β-cell function, the maintenance of glycaemic homeostasis, and the regulation of pancreatic innate immune responses and pancreas development. Another article by Deng et al. investigated the association of Ectodysplasin A, a newly identified hepatokine (13), with type 2 diabetes, and showed for the first time that serum Ectodysplasin A was elevated in patients with type 2 diabetes compared to healthy controls and associated with glycolipid metabolism and insulin resistance.

Finally, the study by Lian et al. explored the relationship of glycaemia with albuminuria, a marker for endothelial dysfunction (14), in the REACTION study. The authors found that the ratio of albumin to creatinine (ACR) and the odds of albuminuria (defined as ACR ≥30 mg/g) were increased progressively with increasing haemoglobin A1c (HbA1c) at the turning point of 5.5% in the general population. This observation highlights the importance of strict glycaemic control for preventing diabetic kidney disease (which is featured by albuminuria), and indicates that renal dysfunction might occur prior to the development of clinical diabetes. Findings from this article may also provoke the consideration of new diagnostic cut-off points of prediabetes or diabetes for HbA1c based on the prevalence of different stages of albuminuria rather than the prevalence of retinopathy evaluated by the protocol from the Early Treatment Diabetic Retinopathy Study (15).

In summary, articles in this Research Topic have added important insights into the pathophysiological role of endothelial dysfunction in diabetes and its complications, and may also provide new possible approaches (e.g., lowering circulating Ectodysplasin A) for the prevention and management of these diseases.

## Author Contributions

All authors listed have made a substantial, direct and intellectual contribution to the work, and approved it for publication.

## Funding

This work was partly supported by the Guangdong Basic and Applied Basic Research Foundation (Grant No. 2020A1515111021). Shanhu Qiu has been supported by the “Best Young Scholars” Fellowship from Southeast University. The funders had no roles in the design of the study and collection, analysis, and interpretation of data or in writing the manuscript.

## Conflict of Interest

The authors declare that the research was conducted in the absence of any commercial or financial relationships that could be construed as a potential conflict of interest.

## Publisher’s Note

All claims expressed in this article are solely those of the authors and do not necessarily represent those of their affiliated organizations, or those of the publisher, the editors and the reviewers. Any product that may be evaluated in this article, or claim that may be made by its manufacturer, is not guaranteed or endorsed by the publisher.
